# Feed-Forward Neural Network Prediction of the Mechanical Properties of Sandcrete Materials

**DOI:** 10.3390/s17061344

**Published:** 2017-06-09

**Authors:** Panagiotis G. Asteris, Panayiotis C. Roussis, Maria G. Douvika

**Affiliations:** 1 Computational Mechanics Laboratory, School of Pedagogical and Technological Education, Heraklion, GR, 14121 Athens, Greece; mariadouvika7@gmail.com; 2 Department of Civil and Environmental Engineering, University of Cyprus, 1678 Nicosia, Cyprus; roussis@ucy.ac.cy

**Keywords:** artificial intelligence techniques, artificial neural networks, compressive strength, modulus of elasticity, non-destructive testing (NDT) methods, sandcrete materials, soft-computing techniques, soft sensors, velocity of ultrasonic pulses

## Abstract

This work presents a soft-sensor approach for estimating critical mechanical properties of sandcrete materials. Feed-forward (FF) artificial neural network (ANN) models are employed for building soft-sensors able to predict the 28-day compressive strength and the modulus of elasticity of sandcrete materials. To this end, a new normalization technique for the pre-processing of data is proposed. The comparison of the derived results with the available experimental data demonstrates the capability of FF ANNs to predict with pinpoint accuracy the mechanical properties of sandcrete materials. Furthermore, the proposed normalization technique has been proven effective and robust compared to other normalization techniques available in the literature.

## 1. Introduction

Low-cost buildings have been constructed at numerous different locations across the globe during history. Eco-friendly, low-cost, sustainable construction materials for utilization in civil engineering projects have attracted much attention during the last decades. At present, low-cost construction materials are mainly used in Africa and Asia and involve a wide variety, like gypsum, lime, clay, sand and stabilised soil, rice husk ash, ground granulated blast furnace slag etc., as partial or total replacement of cement, aggregates, and concrete. An average cost reduction of up to 30% as compared to conventional construction methods can be achieved. As reviewed by Bahar et al. [1], different types of earth construction such as soil-based construction blocks have been used in North Africa for centuries, especially in rural regions and in the desert. Soil can be stabilised either by manual compaction, mechanical compaction, or, the addition of natural fibers. However, with the development of masonry and reinforced concrete, soil-based constructions are designed and addressed to poor population and, hence, are of lower quality. This is mainly due to their durability problems such as the lack of water resistance and erosion [1]. However, due to its higher cost and lower thermal performance, much interest is going back to earth construction, which is known for its cheap labour and low cost and comparable thermal insulation characteristics [1]. This was primarily owed to the inefficiencies associated with such materials, mainly in terms of strength and durability (i.e., lack of water resistance and erosion [1]), as well as the high cost associated mainly with masonry construction. Furthermore, materials such as concrete and steel were easier to use in construction and required less skilled labour.

The nature of soils available for such applications is very diverse due to their origin from very different types of geological deposits including several types of clays, sand resources, crushed rock formations, etc. Apart from the inorganic constituents, a very diverse group of organic components can be also identified.

To this end, a promising alternative is soilcrete, a type of non-conventional construction material produced by mixing natural soil, such as natural clay or limestone sand, with a hydraulic binder [2,3,4,5,6]. Soilcrete belongs to a family of concretes that can be used under conditions whereby environmental or economic constraints limit the use of coarse aggregates, as it exploits amounts of proper soils, rocks or even recycled concrete materials that are present in abundance. Its main components are fine aggregate (clay or/and sand), ordinary Portland cement, water at an appropriate ratio and rarely mineral admixtures, thus rendering it a composite material.

Artificial neural networks (ANNs) have emerged over the last decades as an attractive meta-modelling technique applicable to a vast number of scientific fields including material science among others. An important characteristic of ANNs is that they can be used to build soft-sensors, i.e., models with the ability to estimate critical quantities without having to measure them [7]. In particular, such surrogate models can be constructed after a training process with only a few available data, which can be used to predict pre-selected model parameters, reducing the need for time- and money-consuming experiments. Thus far, the literature includes studies in which ANNs were used for predicting the mechanical properties of concrete materials [8,9,10,11,12,13,14,15,16]. In their study Asteris et al. [16] used ANNs to estimate the compressive strength of self-compacting concrete through a training process involving as input parameters the eleven parameters of synthesis with output parameter the value of the compressive strength. Moreover, similar methods such as fuzzy logic and genetic algorithms have also been used for modelling the compressive strength of concrete materials [17,18,19,20,21,22,23,24]. A detailed state-of-the-art report can be found in [25,26,27,28].

The mechanical properties of sandcrete materials exhibit a strong nonlinear nature derived from the parameters involved in their composition; it is this nonlinear behaviour that makes the development of an analytical formula for the prediction of the mechanical properties using deterministic methods a rather difficult task. In this work, the modelling of the mechanical characteristics of soilcrete materials has been investigated in-depth using soft-computing techniques such as artificial intelligence (AI) techniques. In particular, this study investigates the application of Artificial Neural Networks (ANNs) models for the prediction of the 28-day compressive strength and the modulus of elasticity of sandcrete materials, using the four parameters of synthesis and the value of the ultrasonic velocity as input parameters.

## 2. Significance of the Subject

Much effort in recent years involves the development of reliable methods for the assessment of the vulnerability of existing structures by means of the accurate estimation of the in-situ mechanical characteristics of materials used. The estimation of the mechanical characteristics of concrete has been traditionally carried out through either destructive or non-destructive methods. The greatest disadvantages of the conventional destructive method of extracting cylindrical specimens (cores) to evaluate the mechanical properties of concrete include cost of intervention, damage caused during the execution of the test, and limited number of extracted specimens, which in turn affects negatively the reliability of results.

On the other hand, non-destructive methods, such as the ultrasonic pulse velocity method, provide a simple, inexpensive method to approximate the concrete properties. Based on the measured ultrasonic velocity, a number of analytical expressions for estimating the compressive strength and dynamic modulus of elasticity of concrete have been proposed in the literature [29,30,31,32,33,34]. Nevertheless, the relatively low correlation between the estimated values with the experimental results, highlights the need to develop new methods for the reliable assessment of these important properties.

To this end, soft-computing techniques, such as the Artificial Neural Networks (ANN), can contribute as a feasible tool for the estimation of the mechanical properties of concrete [10,35,36,37,38]. In this study, Artificial Neural Networks have been developed for the prediction of the compressive strength and modulus of elasticity of sandcrete materials, using as input parameters the mix compositions and the value of ultrasonic velocity, while as output parameter the value of compressive strength or the value of modulus of elasticity.

## 3. Artificial Neural Networks

This section summarizes the mathematical and computational aspects of artificial neural networks. In general, ANNs are information-processing models configured for a specific application through a training process. A trained ANN maps a given input onto a specific output, and thereby can be considered to be similar to a response surface method. The main advantage of a trained ANN over conventional numerical analysis procedures (e.g., regression analysis) is that the results can be produced with much less computational effort [16,25,39,40,41,42,43,44,45].

### 3.1. General

The concept of an artificial neural network is based on the concept of the biological neural network of the human brain. The basic building block of the ANN is the artificial neuron, which is a mathematical model trying to mimic the behaviour of the biological neuron. Information is passed into the artificial neuron as input and processed with a mathematical function leading to an output that determines the behaviour of the neuron (similar to fire-or-not situation for the biological neuron). Before the information enters the neuron, it is weighted in order to approximate the random nature of the biological neuron. A group of such neurons consists of an ANN in a manner similar to biological neural networks. In order to set up an ANN, one needs to define: (i) the architecture of the ANN; (ii) the training algorithm, which will be used for the ANN learning phase; and (iii) the mathematical functions describing the mathematical model. The architecture or topology of the ANN describes the way the artificial neurons are organized in the group and how information flows within the network. For example, if the neurons are organized in more than one layers, then the network is called a multilayer ANN. Regarding the training phase of the ANN, it can be considered as a function minimization problem, in which the optimum value of weights need to be determined by minimizing an error function. Depending on the optimization algorithms used for this purpose, different types of ANNs exist. Finally, the two mathematical functions that define the behaviour of each neuron are the summation function and the activation function. In the present study, we use a back-propagation neural network (BPNN), which is described in the next section.

### 3.2. Architecture of BPNN

**Figure 1:**
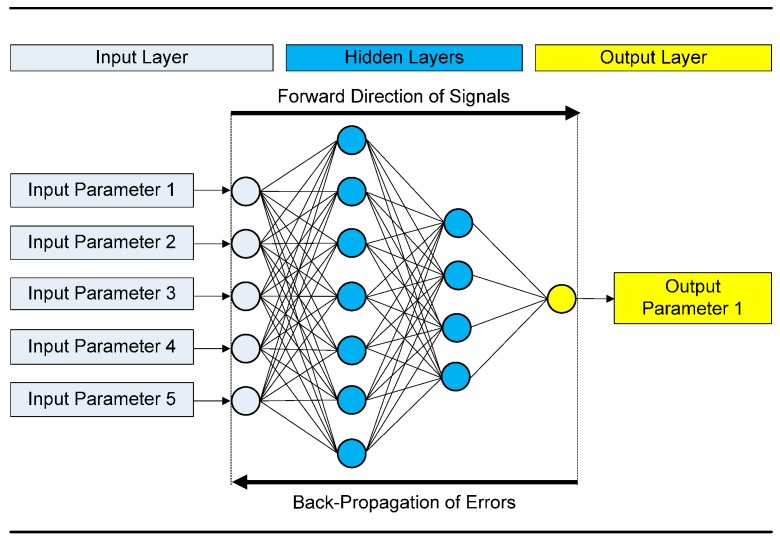
Architecture of a 5-7-4-1 BPNN model.

A BPNN is a feed-forward, multilayer network [39], i.e., information flows only from the input towards the output with no feedback loops, and the neurons of the same layer are not connected to each other, but they are connected with all the neurons of the previous and subsequent layer. A BPNN has a standard structure that can be written as:

(1) $$N-H_{1}-H_{2}-\ldots -H_{NHL}-M$$

where N is the number of input neurons (input parameters);$\;H_{i}$ is the number of neurons in the *i*-th hidden layer for i=1, …,NHL; NHL is the number of hidden layers; and M is the number of output neurons (output parameters). Figure 1 depicts an example of a BPNN composed of an input layer with five neurons, two hidden layers with seven and four neurons, respectively, and an output layer with 1 neuron, i.e., a 5-7-4-1 BPNN.

**Figure 2:**
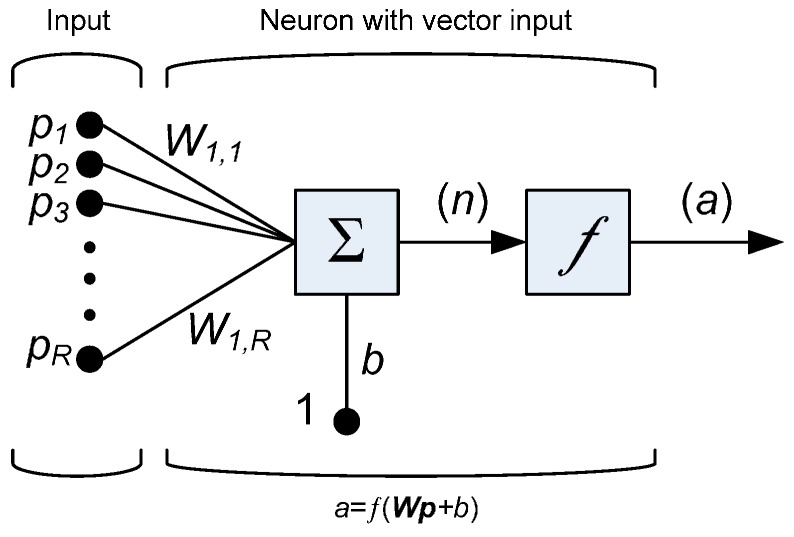
A neuron with a single R-element input vector.

A notation for a single node (with the corresponding R-element input vector) of a hidden layer is presented in Figure 2.

For each neuron *i*, the individual element inputs $p_{1},\;\ldots ,p_{R}$ are multiplied by the corresponding weights $w_{i,1},\;\ldots ,w_{i,R}$ and the weighted values are fed to the junction of the summation function, in which the dot product (W·p) of the weight vector $W=\left[w_{i,1},\;\ldots ,\;w_{i,R}\right]$ and the input vector $p={\left[p_{1},\;\ldots ,p_{R}\right]}^{T}$ is generated. The threshold *b* (bias) is added to the dot-product forming the net input n, which is the argument of the transfer function *f*:

(2) $$n=W\cdot p=w_{i,1}p_{1}+w_{i,2}p_{2}+\ldots +w_{i,R}p_{R}+b$$

The choice of the transfer (or activation) function *f* may strongly influence the complexity and performance of the ANN. Although sigmoidal transfer functions are the most commonly used, one may use different type of functions. Previous studies [46,47] have proposed a large number of alternative transfer functions. In the present study, the Logistic Sigmoid and the Hyperbolic Tangent transfer functions were found to be appropriate for the problem investigated. During the training phase, the training data are fed into the network which tries to create a mapping between the input and the output values. This mapping is achieved by adjusting the weights in order to minimise the following error function:

(3) $$E=\sum^{\text{​}}{\left(x_{i}-y_{i}\right)}^{2}$$

where $x_{i}$ and $y_{i}$ are the measured value and the prediction of the network, respectively, within an optimization framework. The training algorithm used for the optimization plays a crucial role in building a quality mapping, thus an exhaustive investigation was performed in order to find the most suitable for this problem. The most common method used in the literature is the back-propagation technique, in which, as stated by its name, the information propagates to the network in a backward manner in order to adjust the weights and minimize the error function. To adjust the weights properly, a general method called gradient descent is applied, in which the gradients of the error function with respect to the network weights is calculated. Further discussion on the training algorithms is given in the numerical example section.

## 4. Results and Discussion

This section presents the process for tuning optimum ANNs used for the prediction of the 28-day compressive strength and the modulus of elasticity of sandcrete materials, based on experimental data available in the literature.

### 4.1. Experimental

**Figure 3:**
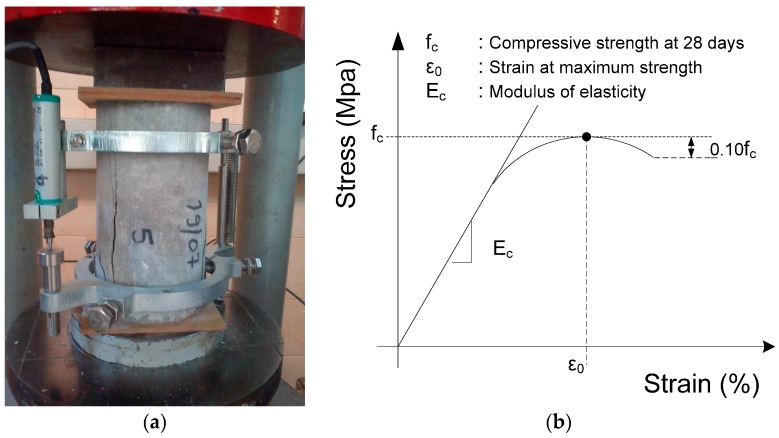
(**a**) Uniaxial compression test setup of sandcrete specimen; (**b**) Stress-strain curves.

A detailed description of the experimental set-up is given by Kolovos [2,3,4]. Data and results within the provided research database (Table 1) present the measured compressive strength $\left(f_{c}\right)$ at 28 days, as well as the modulus of elasticity (Figure 3) of a large set of cylindrical specimens (134) with a height-to-diameter (*h*/*d*) ratio equal to 2 (*h*/*d* = 2) which have been tested under uniaxial compression. For each sample, four to eight soilcrete specimens were tested and the mean value of these measurements is presented in Table 1 [48]. Before the uniaxial compression test, the value of ultrasonic velocity of all specimens has been measured using the widely-adopted TICO (testing instrument of concrete) ultrasonic instrument.

The samples of the soilcrete material consisted of different contents of crushed quarry sand of a limestone origin (CQLS) as replacement of the aggregate phase and high purity commercial metakaolin (MK) supplied by Imerys Minerals (Roswell, GA, USA), added at variable contents as a mineral additive to the ordinary Portland cement-based binder mix at different water/binder ratio values (W/B). Batches of samples with CQLS were produced, by mixing 50 and 70% w/w CQLS (in the dry mix) with 50 and 30% w/w binder at 3 different W/B ratio values of 0.4, 0.7 and 1.0. High workability and optimal flow properties for samples with W/B equal to 0.4 were achieved by the addition of 2.0% w/w (of the cementitious materials) common superplasticizer (SP) (CHEM SLP P), manufactured by Domylco Ltd. (Athens, Greece), as listed in Table 1.

Three categories of binders (B), variable in synthesis, were investigated; the first that consisted of 100% w/w CEM I 42.5 N Portland cement (PC) was used as reference, the second produced by mixing 90% w/w PC and 10% w/w metakaolin (MK) in the dry mix, and the third by mixing 80% w/w PC and 20% w/w MK (in the dry mix) as partial replacement. Homogeneity of all three categories of blends was reached after mixing MK and PC without further grinding in a laboratory swing mill for 1 h. Mixing of the binder-CQLS mixtures with tap water at 20 °C was conducted in an 80 L capacity laboratory mixer appropriate for concrete production. After casting the molds were covered to minimize water evaporation, stripped after 24 h and the specimens were immersed in lime-saturated water at 20 °C, until testing. A detailed and in-depth description of the experimental set-up can be found in the above-mentioned references.

Each input training vector p is of dimension 1 × 5 and consists of the values of the four parameters of synthesis and the value of the ultrasonic velocity (R = 5), namely the water-to-binder ratio (W/B), the metakaolin addition (MK), the binder (B), the superplasticizer (SP), and the ultrasonic velocity (UV). The corresponding output training vectors are of dimension 1 × 1 and consist, in the first case, of the value of the 28 days compressive strength and, in the second case, of the value of the modulus of elasticity of the sandcrete specimens. Their mean values together with the minimum and maximum values are listed in Table 2.

### 4.2. Normalization of Data

As mentioned previously, the normalization of the input and output parameters has a significant impact on the ANN training. In the present study, during the pre-processing stage, the Min-Max [49] and the ZScore normalization methods have been used. In particular, the five input parameters (Table 1), as well as the output parameters of the 28-day compressive strength and the modulus of elasticity have been normalized using the Min-Max normalization method. As stated in Iruansi et al. [50], in order to avoid problems associated with low learning rates of the ANN, the normalization of the data should be made within a range defined by appropriate upper and lower limit values of the corresponding parameter. In this work, the input and output parameters have been normalized in the range [0,1] and [−1,1], respectively. Moreover, in this work a recently proposed transformation technique called Central has been applied [51], in which the origin of the training data is shifted to the centre of the data with the following formula:

(4) $$z_{i}=x_{i}-\frac{\max\left(x\right)+\min\left(x\right)}{2}$$

where $x\;\left(x_{1},\;x_{2},\;\ldots ,x_{n}\right)$ are the original data and $z_{i}$ is the i-th transformed data.

### 4.3. BPNN Model Development

In this work, a large number of different BPNN models have been developed and implemented in four different computers in order to investigate the sensitivity of the ANN results to the very nature of the floating-point arithmetic of each computer. Each one of these ANN models was trained over 90 data-points out of the total of 134 data-points, (66.86% of the total number) and the validation and testing of the trained ANN were performed with the remaining 44 data-points. More specifically, 22 data-points (16.57%) were used for the validation of the trained ANN and 22 (16.57%) data-points were used for the testing (estimating the Pearson’s correlation coefficient R). The parameters used for the ANN training are summarized as follows.

After a detailed and in-depth investigation among a plethora of training algorithms, the Levenberg-Marquardt algorithm [52] has been used as the optimum training algorithm for the ANN models. The development and training of the ANNs occurs with a number of hidden layers ranging from one to two and with a number of neurons ranging from one to 30 for each hidden layer. Each one of the ANNs is developed and trained for a number of different activation functions, such as the Logistic Sigmoid and the Hyperbolic Tangent functions.

In order to achieve a fair comparison of the various ANNs, the datasets used for their training are manually divided by the user into training, validation, and testing sets using appropriate indices to state whether the data belongs to the training, validation or testing set. In the general case, the division of the datasets into the three groups is made randomly.

Based on the above, a total of 372,000 different BPNN models have been developed and investigated in order to find the optimum NN model for the prediction of the compressive strength (186,000) and the modulus of elasticity (186,000) of sandcrete materials.

**Figure 4:**
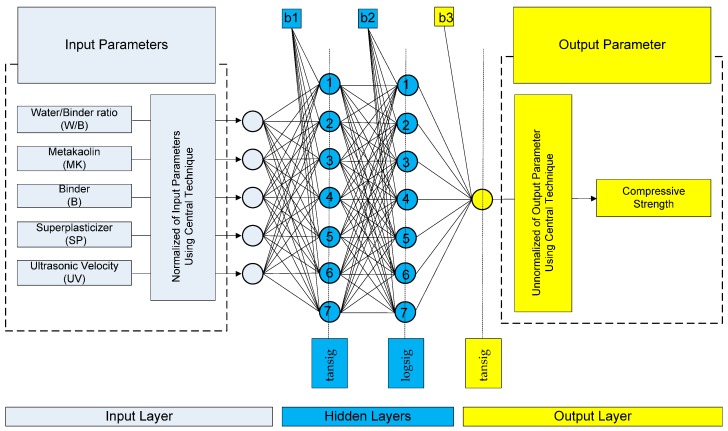
Architecture of the optimum with two hidden layers 5-7-7-1 BPNN model for the case of compressive strength of sandcrete materials based on Pearson’s correlation coefficient R.

**Figure 5:**
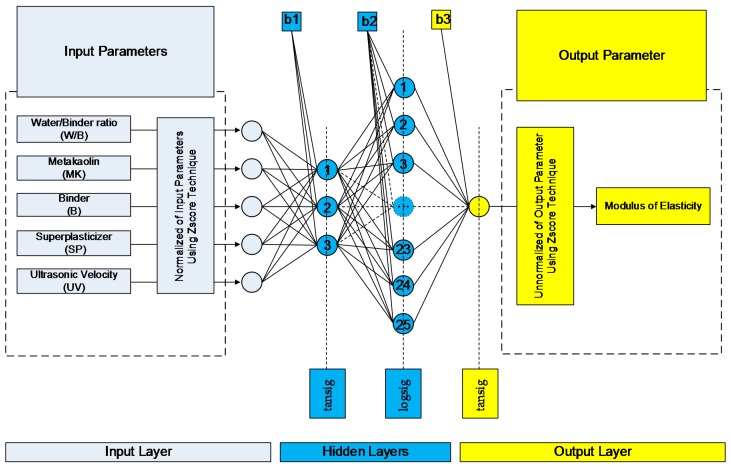
Architecture of the optimum with two hidden layers 5-3-25-1 BPNN model for the case of modulus of elasticity of sandcrete materials based on Pearson’s correlation coefficient R.

The developed ANN models were sorted in a decreasing order based on the Pearson’s correlation coefficient value. The architectures of the top twenty models are presented in Table 3 for the case of compressive strength and in Table 4 for the case of modulus of elasticity. Based on these results, the optimum BPNN model for the prediction of the compressive strength is that of 5-7-7-1 (Figure 4) with Pearson’s correlation coefficient R equal to 0.99001, while for the prediction of the modulus of elasticity is that of 5-3-25-1 (Figure 5) with Pearson’s correlation coefficient R equal to 0.988105. Figure 4 and Figure 5 present in detail the architectures of the two optimum NNs for the compressive strength and the modulus of elasticity. Furthermore, these figures depict the methodology followed to achieve the two NN models. Based on the information provided in these figures and the final values of weights presented in the next section, one could readily develop a simple code to be used as a tool both for the practicing engineers, as well as researchers in the field, for the prediction of the mechanical properties of sandcrete materials.

**Figure 6:**
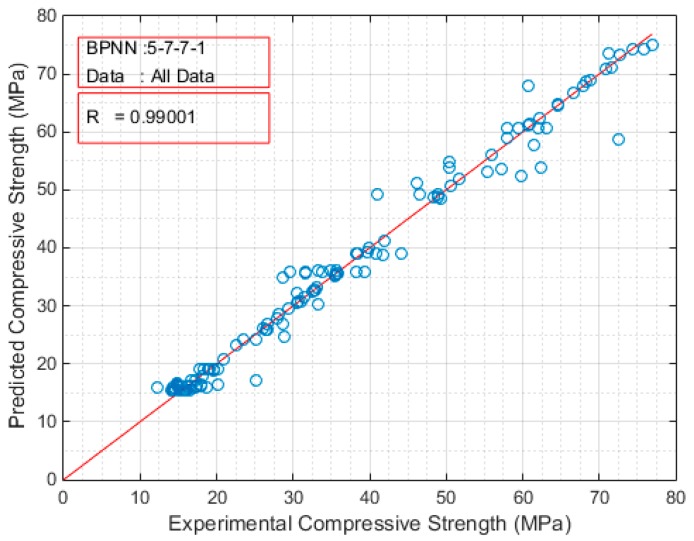
The Pearson’s correlation coefficient R of the experimental and predicted compressive strength for the best with two hidden layers BPNN (5-7-7-1).

**Figure 7:**
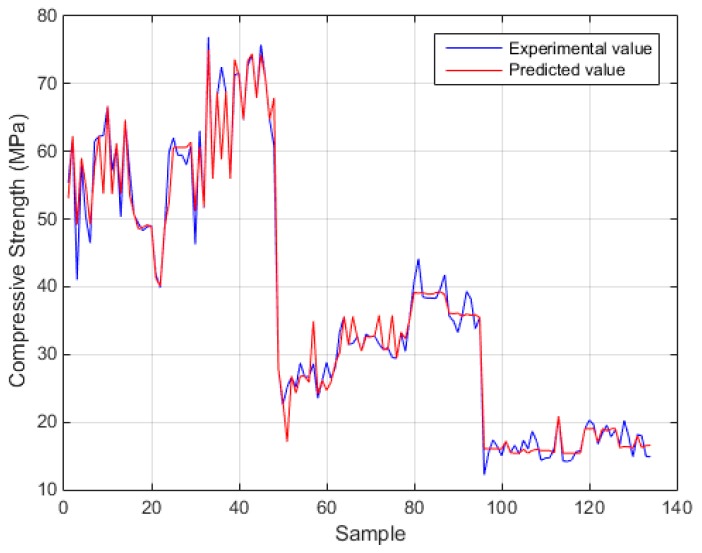
Experimental vs predicted values of compressive strength for the best with two hidden layers BPNN (5-7-7-1).

**Figure 8:**
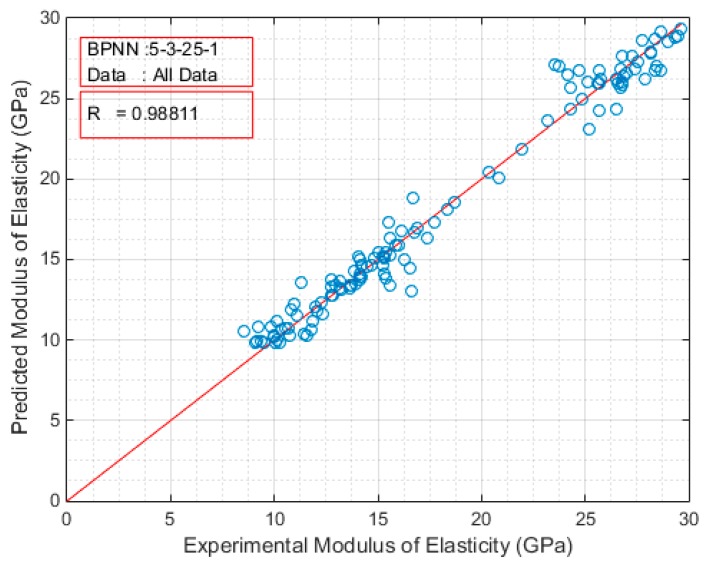
The Pearson’s correlation coefficient R of the experimental and predicted modulus of elasticity for the best with two hidden layers BPNN (5-3-25-1).

**Figure 9:**
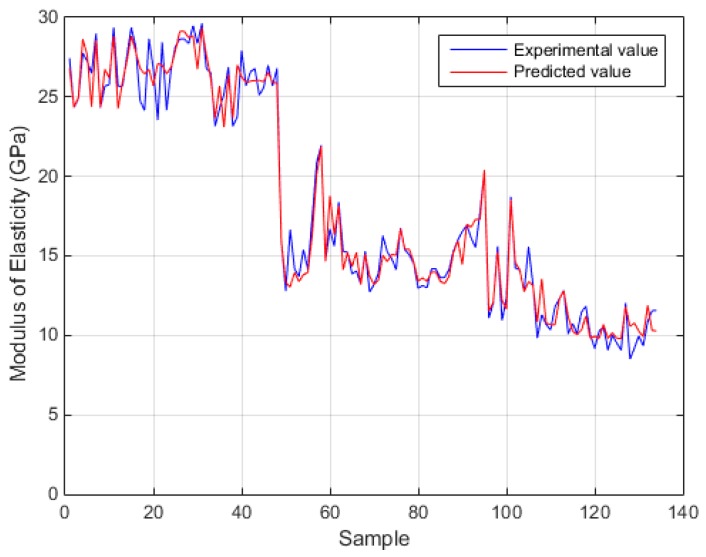
Experimental vs predicted values of modulus of elasticity for the best with two hidden layers BPNN (5-3-25-1).

Figure 6 and Figure 7 depict the comparison of the exact experimental values with the predicted values of the optimum BPNN model with topology 5-7-7-1 for the case of compressive strength, while Figure 8 and Figure 9 for the case of modulus of elasticity of sandcrete materials. These results clearly show that the 28-day compressive strength and the modulus of elasticity of sandcrete material predicted from the multilayer feed-forward neural network are very close to the experimental results.

### 4.4. Final Values of Weights of the FF-NN Model

It is common practice in the majority of published papers on NN models, for authors to present the architecture of the optimum NN model without providing any information regarding the final values of NN weights. Admittedly, this practice has very little value for other researchers and practicing engineers. In order to be useful, a proposed NN architecture should be accompanied by the (quantitative) values of weights. In such a case, the NN model can be readily implemented in an MS-Excel spreadsheet, thus available to anyone interested in the problem of modelling.

**Figure 10:**
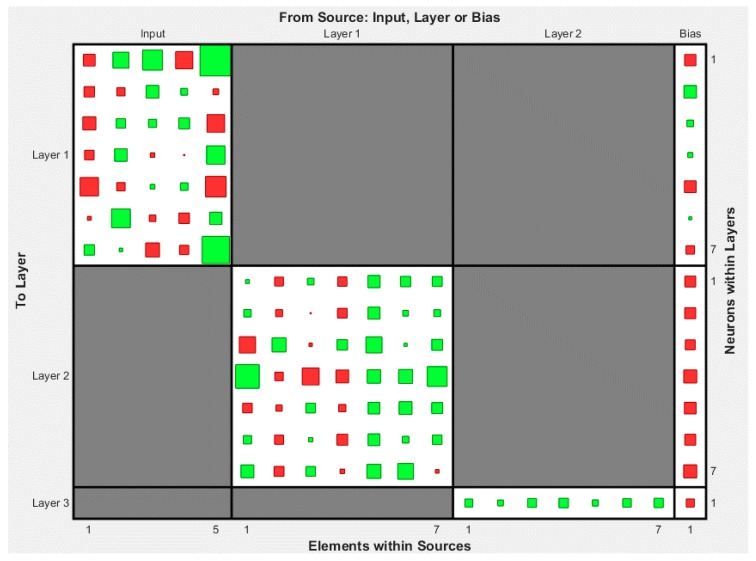
Final Weights and Bias Values of the optimum FF-NN model 5-7-7-1 for the case of compressive strength of sandcrete materials (The values are presented in Table A1 of Appendix A).

**Figure 11:**
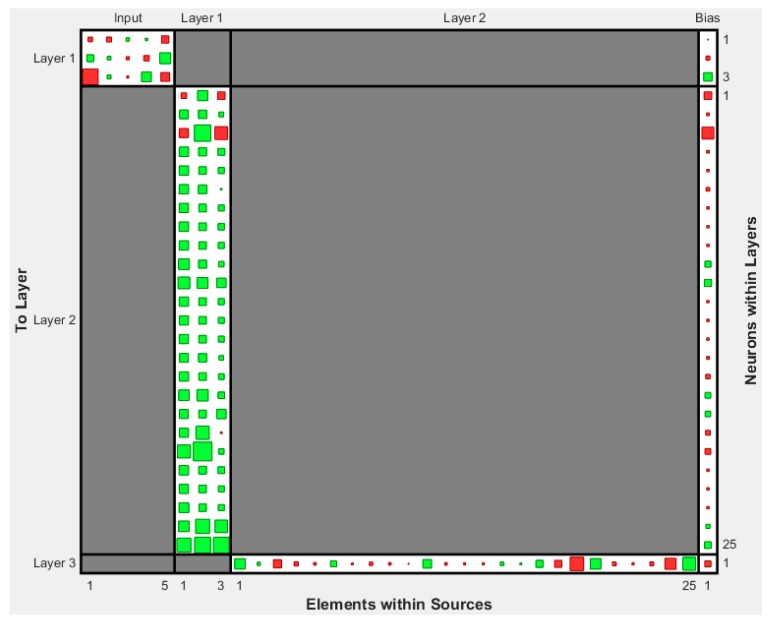
Final Weights and Bias Values of the optimum FF-NN model 5-3-25-1 for the case of modulus of elasticity of sandcrete materials (The values are presented in Table A2 of Appendix A).

In this study, the final values of weights and biases for both cases of compressive strength and modulus of elasticity are explicitly reported in Figure 10 and Figure 11 and Table A1 and Table A2 of the Appendix A. By employing the properties defined in Table 1 and using the reported values of weights and biases, one can easily build an ANN-based estimator for the compressive strength and the modulus of elasticity of sandcrete materials.

## 5. Discussion

Based on the above optimum NN models for the prediction of the compressive strength and modulus elasticity of sandcrete materials, the following can be stated:

1. Among the training algorithms available in the literature, the best by far ANN prediction of the sandcrete compressive strength was achieved by using the Levenberg-Marquardt algorithm.
2. Different optimum ANN architectures were found in different computers, which means that the computational environment affects the procedure of ANN training, and subsequently its performance. This can be attributed to the fact that the tested algorithms ultimately rely on basic arithmetic operations that can yield different results, when performed in different environments, due to the very nature of floating-point arithmetic.
3. The recently-proposed formula for the normalization of data proved effective and robust compared to available ones.
4. For the top twenty models the optimum number of hidden layers was found to be two. This is an indication that the complexity of the problem cannot be dealt with effectively with a single hidden layer.
5. The best activation functions corresponding to all of the top-twenty best NN models, both for the case of compressive strength and modulus of elasticity, are the same, namely Hyperbolic tangent sigmoid transfer function for the first hidden layer, Log-sigmoid transfer function for the second hidden layer, and also Hyperbolic tangent sigmoid transfer function for the output layer.

## 6. Conclusions

This study investigated the application of Artificial Neural Networks (ANNs) models for the prediction of the mechanical properties of sandcrete materials. The comparison of the derived results with the experimental findings demonstrates the effectiveness of ANNs to build soft sensors with the ability to predict in a reliable manner the mechanical properties of sandcrete materials. Moreover, the results obtained using the proposed technique for pre-processing the data were favourably compared to the results obtained by other known normalization techniques available in the literature. This fact demonstrates the need for further research on data pre-processing, prior to the use of the data toward the training and the development of ANNs, taking into account the present-day limitations and constraints in this promising research area.

## Figures and Tables

**Table 1:** Experimental data/results and input and output parameters of BPNNs.

Sample	Input					Output		Comments ^1^
	W/B Ratio	MK (% w/w in the Dry Mix)	B (% w/w in the Dry Mix)	SP (% w/w of the Cementitious Materials)	Ultrasonic Velocity (m/s)	Compressive Strength (MPa)	Modulus of Elasticity (GPa)	
1	0.40	0	50	2	4070.00	55.35	27.442	T
2	0.40	0	50	2	4016.67	62.25	24.325	T
3	0.40	0	50	2	4053.33	41.04	24.875	V
4	0.40	0	50	2	4100.00	58.00	27.754	T
5	0.40	0	50	2	4076.67	50.35	27.249	T
6	0.40	0	50	2	4040.00	46.48	26.476	Test
7	0.40	0	50	2	4090.00	61.49	28.976	T
8	0.40	0	50	2	4016.67	62.25	24.325	T
9	0.40	0	30	2	4006.67	62.35	25.690	V
10	0.40	0	30	2	4080.00	66.72	25.765	T
11	0.40	0	30	2	4040.00	57.17	29.371	T
12	0.40	0	30	2	4100.00	60.79	25.679	Test
13	0.40	0	30	2	4000.00	50.36	25.650	T
14	0.40	0	30	2	4070.00	64.64	27.577	T
15	0.40	0	30	2	4040.00	57.17	29.371	V
16	0.40	0	30	2	4063.33	50.66	28.145	T
17	0.40	5	50	2	3913.33	49.36	24.745	T
18	0.40	5	50	2	3931.67	48.30	24.160	Test
19	0.40	5	50	2	3916.67	48.86	28.668	T
20	0.40	5	50	2	3980.00	49.01	26.747	T
21	0.40	5	50	2	3840.00	41.86	23.543	V
22	0.40	5	50	2	3900.00	39.87	28.434	T
23	0.40	5	50	2	3931.67	48.30	24.160	T
24	0.40	5	50	2	3810.00	59.82	26.746	Test
25	0.40	3	30	2	4090.00	62.01	28.182	T
26	0.40	3	30	2	4053.33	59.44	28.644	T
27	0.40	3	30	2	4053.33	59.44	28.644	V
28	0.40	3	30	2	4070.00	58.03	28.360	T
29	0.40	3	30	2	4003.33	60.87	29.478	T
30	0.40	3	30	2	3966.67	46.26	28.360	Test
31	0.40	3	30	2	4023.33	63.05	29.625	T
32	0.40	3	30	2	3986.67	51.67	26.791	T
33	0.40	10	50	2	3926.67	76.90	26.513	V
34	0.40	10	50	2	3831.67	56.03	23.159	T
35	0.40	10	50	2	3763.33	68.21	24.276	T
36	0.40	10	50	2	3810.00	72.48	25.168	Test
37	0.40	10	50	2	3873.33	68.86	26.876	T
38	0.40	10	50	2	3831.67	56.03	23.159	T
39	0.40	10	50	2	3746.67	71.26	23.733	V
40	0.40	10	50	2	3756.67	71.57	27.914	T
41	0.40	6	30	2	3886.67	64.65	25.695	T
42	0.40	6	30	2	3820.00	72.68	26.582	Test
43	0.40	6	30	2	3906.67	74.34	26.781	T
44	0.40	6	30	2	3880.00	67.92	25.120	T
45	0.40	6	30	2	3903.33	75.77	25.578	V
46	0.40	6	30	2	3863.33	70.94	26.992	T
47	0.40	6	30	2	3886.67	64.65	25.695	T
48	0.40	6	30	2	3890.00	60.81	26.798	Test
49	0.70	0	50	0	3523.33	27.87	15.884	T
50	0.70	0	50	0	3353.33	22.53	12.785	T
51	0.70	0	50	0	3333.33	25.16	16.654	V
52	0.70	0	50	0	3381.67	26.68	14.166	T
53	0.70	0	50	0	3356.67	25.18	13.710	T
54	0.70	0	50	0	3376.67	28.75	15.392	Test
55	0.70	0	50	0	3381.67	26.68	14.166	T
56	0.70	0	30	0	3486.67	26.72	17.402	T
57	0.70	0	30	0	3670.00	28.63	20.870	V
58	0.70	0	30	0	3536.67	23.53	21.957	T
59	0.70	0	30	0	3343.33	26.07	14.697	T
60	0.70	0	30	0	3516.67	28.83	16.694	Test
61	0.70	0	30	0	3486.67	26.44	15.622	T
62	0.70	0	30	0	3436.67	28.06	18.379	T
63	0.70	0	30	0	3413.33	33.32	15.280	V
64	0.70	5	50	0	3303.33	35.60	15.233	T
65	0.70	5	50	0	3406.67	31.48	13.865	T
66	0.70	5	50	0	3303.33	31.61	14.062	Test
67	0.70	5	50	0	3333.33	32.59	13.197	T
68	0.70	5	50	0	3533.33	30.51	15.296	T
69	0.70	5	50	0	3383.33	32.99	12.730	V
70	0.70	5	50	0	3333.33	32.59	13.197	T
71	0.70	5	50	0	3373.33	32.71	13.913	T
72	0.70	3	30	0	3473.33	31.53	16.269	Test
73	0.70	3	30	0	3530.00	30.69	15.240	T
74	0.70	3	30	0	3516.67	31.00	14.834	T
75	0.70	3	30	0	3473.33	29.55	14.138	V
76	0.70	3	30	0	3420.00	29.43	16.757	T
77	0.70	3	30	0	3493.33	33.11	15.356	T
78	0.70	3	30	0	3500.00	30.44	15.064	Test
79	0.70	3	30	0	3446.67	35.51	14.515	T
80	0.70	10	50	0	3386.67	40.78	12.960	T
81	0.70	10	50	0	3396.67	44.13	13.138	V
82	0.70	10	50	0	3386.67	38.48	12.989	T
83	0.70	10	50	0	3416.67	38.33	14.179	T
84	0.70	10	50	0	3416.67	38.33	14.179	Test
85	0.70	10	50	0	3386.67	38.28	13.626	T
86	0.70	10	50	0	3373.33	39.71	13.640	T
87	0.70	10	50	0	3426.67	41.75	14.144	V
88	0.70	6	30	0	3473.33	35.68	15.308	T
89	0.70	6	30	0	3466.67	34.94	16.008	T
90	0.70	6	30	0	3480.00	33.27	16.529	Test
91	0.70	6	30	0	3423.33	35.82	16.929	T
92	0.70	6	30	0	3456.67	39.31	16.124	T
93	0.70	6	30	0	3440.00	38.16	15.525	V
94	0.70	6	30	0	3446.67	33.79	17.725	T
95	0.70	6	30	0	3400.00	35.49	20.365	T
96	1.00	0	50	0	2996.67	12.21	11.080	Test
97	1.00	0	50	0	3076.67	15.41	12.005	T
98	1.00	0	50	0	3216.67	17.39	15.598	T
99	1.00	0	50	0	3086.67	16.39	10.943	V
100	1.00	0	50	0	3026.67	15.05	12.352	T
101	1.00	0	30	0	3430.00	17.21	18.732	T
102	1.00	0	30	0	3233.33	15.52	14.212	Test
103	1.00	0	30	0	3173.33	16.56	14.149	T
104	1.00	0	30	0	3083.33	15.28	12.740	T
105	1.00	5	50	0	3163.33	17.32	15.575	V
106	1.00	5	50	0	3230.00	16.03	13.224	T
107	1.00	5	50	0	3053.33	18.64	9.836	T
108	1.00	5	50	0	3180.00	17.20	11.299	Test
109	1.00	5	50	0	3040.00	14.37	10.686	T
110	1.00	5	50	0	2933.33	14.67	10.341	T
111	1.00	5	50	0	3010.00	14.74	11.803	V
112	1.00	3	30	0	3116.67	16.13	12.304	T
113	1.00	3	30	0	3350.00	20.84	12.832	T
114	1.00	3	30	0	3130.00	14.28	10.094	Test
115	1.00	3	30	0	2993.33	14.16	10.735	T
116	1.00	3	30	0	3180.00	14.42	10.125	T
117	1.00	3	30	0	3006.67	15.60	11.474	V
118	1.00	3	30	0	3063.33	15.74	11.834	T
119	1.00	10	50	0	2906.67	19.00	10.029	T
120	1.00	10	50	0	2983.33	20.26	9.171	Test
121	1.00	10	50	0	2896.67	19.60	10.271	T
122	1.00	10	50	0	3060.00	16.73	10.539	T
123	1.00	10	50	0	2890.00	18.38	9.072	V
124	1.00	10	50	0	3023.33	19.54	10.013	T
125	1.00	10	50	0	2930.00	17.85	9.515	T
126	1.00	10	50	0	2896.67	18.82	9.079	Test
127	1.00	6	30	0	3070.00	16.67	12.034	T
128	1.00	6	30	0	3003.33	20.24	8.509	T
129	1.00	6	30	0	3013.33	17.89	9.197	V
130	1.00	6	30	0	3086.67	14.86	9.972	T
131	1.00	6	30	0	2926.67	18.16	9.338	T
132	1.00	6	30	0	3046.67	17.95	10.852	Test
133	1.00	6	30	0	2986.67	14.92	11.560	T
134	1.00	6	30	0	2983.33	14.89	11.570	T

^1^ T: Training Data; V: Validation Data; Test: Test Data.

**Table 2:** The input and output parameters used in the development of BPNNs.

Code	Parameter Type	Variable	Data Used in NN Models		
			Minimum	Average	Maximum
01	Input	Water-to-binder ratio	0.40	0.68	1.00
02	Input	Metakaolin	0.00	4.24	10.00
03	Input	Binder	30.00	40.00	50.00
04	Input	Superplasticizer	0.00	0.72	2.00
05	Input	Ultrasonic velocity (m/s)	2890.00	3511.82	4100.00
06	Output	Compressive strength (MPa)	12.21	37.46	76.90
07	Output	Modulus of Elasticity (GPa)	8.51	18.20	29.62

**Table 3:** Ranking of the top twenty best architectures of BPNNs (Compressive strength).

Ranking	Computer	Preprocess	Cost Function ^1^	Training Functions ^2^	Initial Weights	Architecture (Code)	Pearson’s R	Number of Epochs
1	C02	Central	MSE	T-L-T	−0.10	5-7-7-1	0.990015	180
2	C04	MinMax [0,1]	MSE	T-L-T	−0.70	5-30-7-1	0.989846	218
3	C03	MinMax [0,1]	MSE	T-L-T	0.90	5-6-24-1	0.989165	215
4	C03	MinMax [−1,1]	SSE	T-L-T	−0.70	5-12-14-1	0.988943	168
5	C04	MinMax [−1,1]	MSE	T-L-T	0.10	5-6-24-1	0.988755	213
6	C04	MinMax [−1,1]	SSE	T-L-T	0.10	5-13-30-1	0.988752	213
7	C03	MinMax [0,1]	MSE	T-L-T	0.90	5-6-27-1	0.988698	215
8	C02	MinMax [0,1]	MSE	T-L-T	−0.90	5-4-3-1	0.988587	211
9	C02	Central	MSE	T-L-T	0.10	5-30-3-1	0.988582	180
10	C02	Central	SSE	T-L-T	−0.70	5-30-12-1	0.988463	225
11	C04	MinMax [−1,1]	MSE	T-L-T	0.90	5-6-30-1	0.988389	213
12	C01	ZScore	MSE	T-L-T	0.30	5-11-26-1	0.988233	227
13	C02	Central	MSE	T-L-T	0.90	5-14-29-1	0.988226	180
14	C03	NoPreprocess	SSE	T-L-T	−0.70	5-22-15-1	0.988204	202
15	C04	MinMax [−1,1]	SSE	T-L-T	0.10	5-11-27-1	0.988033	213
16	C03	MinMax [−1,1]	SSE	T-L-T	0.10	5-11-27-1	0.988033	168
17	C03	Central	SSE	T-L-T	−0.50	5-6-3-1	0.988025	250
18	C03	MinMax [−1,1]	MSE	T-L-T	0.30	5-5-25-1	0.987961	168
19	C03	ZScore	MSE	T-L-T	−0.30	5-5-14-1	0.987953	193
20	C01	ZScore	MSE	T-L-T	0.70	5-9-6-1	0.987947	204

^1^ MSE: Mean Square Error; SSE: Mean Square Error; ^2^ T: Hyperbolic tangent sigmoid transfer function (tansig); L: Log-sigmoid transfer function (logsig).

**Table 4:** Ranking of the top twenty best architectures of BPNNs (Modulus of elasticity).

Ranking	Computer	Preprocess	Cost Function ^1^	Training Functions ^2^	Initial Weights	Architecture (Code)	Pearson’s R	Number of Epochs
1	C02	ZScore	MSE	T-L-T	0.90	5-3-25-1	0.988105	191
2	C03	ZScore	MSE	T-L-T	0.10	5-5-20-1	0.988094	148
3	C04	ZScore	MSE	T-L-T	0.70	5-5-28-1	0.987907	241
4	C03	ZScore	MSE	T-L-T	0.70	5-5-28-1	0.987907	148
5	C03	Central	SSE	T-L-T	−0.70	5-6-27-1	0.987766	185
6	C01	MinMax [−1,1]	MSE	T-L-T	0.10	5-4-13-1	0.987665	174
7	C04	MinMax [−1,1]	MSE	T-L-T	−0.90	5-5-30-1	0.987087	208
8	C02	ZScore	MSE	T-L-T	0.10	5-5-17-1	0.987033	131
9	C01	Central	MSE	T-L-T	−0.70	5-7-15-1	0.986940	153
10	C03	MinMax [−1,1]	MSE	T-L-T	−0.70	5-4-2-1	0.986897	147
11	C01	Central	MSE	T-L-T	0.90	5-25-15-1	0.986894	190
12	C01	MinMax [−1,1]	SSE	T-L-T	0.50	5-18-21-1	0.986871	174
13	C04	MinMax [0,1]	MSE	T-L-T	0.30	5-8-13-1	0.986859	130
14	C02	MinMax [−1,1]	MSE	T-L-T	−0.90	5-4-21-1	0.986707	207
15	C01	NoPreprocess	MSE	T-L-T	0.90	5-20-14-1	0.986605	196
16	C03	ZScore	MSE	T-L-T	−0.10	5-5-10-1	0.986596	240
17	C03	MinMax [0,1]	MSE	T-L-T	−0.90	5-24-7-1	0.986517	181
18	C01	Central	MSE	T-L-T	−0.10	5-5-6-1	0.986478	153
19	C01	Central	MSE	T-L-T	−0.90	5-8-27-1	0.986463	241
20	C03	NoPreprocess	MSE	T-L-T	−0.50	5-8-29-1	0.986440	91

^1^ MSE: Mean Square Error; SSE: Mean Square Error; ^2^ T: Hyperbolic tangent sigmoid transfer function (tansig); L: Log-sigmoid transfer function (logsig).

## Appendix A

**Table A1:** Final Weights and Bias Values of the optimum FF-NN model 5-7-7-1 for the case of compressive strength of sandcrete materials.

**iw{1,1}**							**b{1,1}**
−3.1454	5.7724	8.9543	−6.7047	22.5041			−2.9032
−2.4742	−1.5216	3.6697	1.0200	−0.7726			3.5424
−3.9296	1.9843	1.4874	2.6574	−6.9825			0.9965
−2.0427	3.5493	−0.4779	−0.0410	7.5813			0.5879
−7.6746	−1.5906	0.4895	1.3043	−9.5646			−3.1589
−0.3579	7.9553	−1.0003	−2.5111	3.4250			0.2015
2.4607	0.2907	−4.4205	−1.9332	16.6615			−1.6439
**iw{2,1}**							**b{2,1}**
0.3389	−1.8205	0.9802	−1.9670	3.3510	2.3617	2.2468	−2.7143
1.2257	−1.1260	−0.0406	−2.1345	3.2631	0.7258	1.0570	−2.7688
−6.0180	4.5068	−0.2757	2.4946	5.8850	0.2355	2.6817	−2.2599
12.5716	−1.6461	−6.4402	−3.7017	4.0460	4.3573	8.8892	−3.9000
−2.0902	−0.9018	2.1424	−1.2080	3.0220	3.7258	2.7311	−3.1176
1.5583	−1.8878	0.4010	−2.7070	3.1374	1.0304	2.0414	−2.7023
3.7293	−2.2113	2.1434	−0.4837	4.4761	5.6031	−0.3302	−3.9391
**iw{3,2}**							**b{3,1}**
1.4109	0.7937	1.7777	2.0058	0.8540	1.6295	1.9678	−1.4850

iw{1,1} = Weights values for Input Layer; iw{2,1} = Weights values for First Hidden Layer; iw{3,2} = Weights values for Second Hidden Layer; b{1,1} = Bias values for First Hidden Layer; b{2,1} = Bias values for Second Hidden Layer; b{3,1} = Bias values for Output Layer.

**Table A2:** Final Weights and Bias Values of the optimum FF-NN model 5-3-25-1 for the case of modulus of elasticity of sandcrete materials.

**iw{1,1}**						**b{1,1}**
−0.7749	−0.9128	0.4512	0.2054	−1.9381		0.0422
1.7987	0.4968	−0.3772	−1.0875	4.3027		−0.6290
−8.7504	0.5531	−0.1747	3.4623	−2.8384		2.6341
**iw{2,1}**					**(iw{3,2})’**	**b{2,1}**
−1.1246	3.7720	−2.0741			3.9137	−2.1271
2.8413	2.5212	0.8341			0.3920	−0.3034
−3.0563	9.7899	−5.9379			−2.4649	−5.1664
3.2099	2.3507	1.7487			−0.6487	−0.2885
3.1650	2.0482	1.3610			−0.2791	−0.2298
3.1411	2.7374	0.1086			1.3271	−0.4980
3.0513	2.1228	1.3024			−0.1487	−0.2583
3.2524	2.1063	1.4924			−0.4534	−0.2283
3.1566	2.0521	1.3569			−0.2689	−0.2322
4.1909	2.3250	1.1062			−0.0453	1.2783
4.9801	4.2184	3.2779			2.7336	1.8558
3.1562	2.0523	1.3567			−0.2685	−0.2323
3.0853	2.0961	1.3216			−0.1870	−0.2508
3.0732	2.1052	1.3150			−0.1734	−0.2536
2.8481	2.5424	0.7734			0.4513	−0.3137
3.1216	2.1056	1.2121			0.1486	−0.7483
4.0254	4.6646	1.5502			1.8815	1.2186
2.9450	2.1427	3.2016			−1.8828	1.1351
2.9880	6.4671	−0.1321			−6.6844	−0.8629
6.0950	12.6888	1.0980			4.1027	−1.2059
3.2443	2.2189	1.6067			−0.5419	−0.2548
3.0972	2.0876	1.3278			−0.2005	−0.2479
3.4909	2.0370	1.3709			−0.5782	−0.2315
4.0724	7.2089	5.6700			−4.5069	0.6307
7.0715	9.1544	9.1090			6.0504	1.6593
						**b{3,1}**
						−1.4004

iw{1,1} = Weights values for Input Layer; iw{2,1} = Weights values for First Hidden Layer; iw{3,2} = Weights values for Second Hidden Layer; b{1,1} = Bias values for First Hidden Layer; b{2,1} = Bias values for Second Hidden Layer; b{3,1} = Bias values for Output Layer.

## References

[1] Bahar R., Benazzoug M., Kenai S. (2004). Performance of compacted cement-stabilised soil. Cem. Concr. Compos..

[2] Kolovos K.G., Asteris P.G., Cotsovos D.M., Badogiannis E., Tsivilis S. (2013). Mechanical properties of soilcrete mixtures modified with metakaolin. Constr. Build. Mater..

[3] Kolovos K.G., Asteris P.G., Tsivilis S. (2016). Properties of sandcrete mixtures modified with metakaolin. Eur. J. Environ. Civ. Eng..

[4] Asteris P.G., Kolovos K.G., Athanasopoulou A., Plevris V., Konstantakatos G. (2017). Investigation of the mechanical behaviour of metakaolin-based sandcrete mixtures. Eur. J. Environ. Civ. Eng..

[5] Helson O., Beaucour A.L., Eslami J., Noumowe A., Gotteland P. (2017). Physical and mechanical properties of soilcrete mixtures: Soil clay content and formulation parameters. Constr. Build. Mater..

[6] Kim B., Kim Y. (2017). Strength characteristics of cemented sand–bentonite mixtures with fiber and metakaolin additions. Mar. Georesour. Geotechnol..

[7] Alexandridis A. (2013). Evolving RBF neural networks for adaptive soft-sensor design. Int. J. Neural Syst..

[8] Dias W.P.S., Pooliyadda S.P. (2001). Neural networks for predicting properties of concretes with admixtures. Constr. Build. Mater..

[9] Lee S.C. (2003). Prediction of concrete strength using artificial neural networks. Eng. Struct..

[10] Topçu I.B., Saridemir M. (2008). Prediction of compressive strength of concrete containing fly ash using artificial neural networks and fuzzy logic. Comput. Mater. Sci..

[11] Trtnik G., Kavčič F., Turk G. (2009). Prediction of concrete strength using ultrasonic pulse velocity and artificial neural networks. Ultrasonics.

[12] Waszczyszyn Z., Ziemiański L. (2001). Neural networks in mechanics of structures and materials—New results and prospects of applications. Comput. Struct..

[13] Belalia Douma O., Boukhatem B., Ghrici M., Tagnit-Hamou A. (2016). Prediction of properties of self-compacting concrete containing fly ash using artificial neural network. Neural Comput. Appl..

[14] Mashhadban H., Kutanaei S.S., Sayarinejad M.A. (2016). Prediction and modeling of mechanical properties in fiber reinforced self-compacting concrete using particle swarm optimization algorithm and artificial neural network. Constr. Build. Mater..

[15] Açikgenç M., Ulaş M., Alyamaç K.E. (2015). Using an Artificial Neural Network to Predict Mix Compositions of Steel Fiber-Reinforced Concrete. Arab. J. Sci. Eng..

[16] Asteris P.G., Kolovos K.G., Douvika M.G., Roinos K. (2016). Prediction of self-compacting concrete strength using artificial neural networks. Eur. J. Environ. Civ. Eng..

[17] Baykasoǧlu A., Dereli T.U., Taniş S. (2004). Prediction of cement strength using soft computing techniques. Cem. Concr. Res..

[18] Akkurt S., Tayfur G., Can S. (2004). Fuzzy logic model for the prediction of cement compressive strength. Cem. Concr. Res..

[19] Özcan F., Atiş C.D., Karahan O., Uncuoğlu E., Tanyildizi H. (2009). Comparison of artificial neural network and fuzzy logic models for prediction of long-term compressive strength of silica fume concrete. Adv. Eng. Softw..

[20] Eskandari-Naddaf H., Kazemi R. (2017). ANN prediction of cement mortar compressive strength, influence of cement strength class. Constr. Build. Mater..

[21] Oh T.-K., Kim J., Lee C., Park S. (2017). Nondestructive concrete strength estimation based on electro-mechanical impedance with artificial neural network. J. Adv. Concr. Technol..

[22] Khademi F., Akbari M., Jamal S.M., Nikoo M. (2017). Multiple linear regression, artificial neural network, and fuzzy logic prediction of 28 days compressive strength of concrete. Front. Struct. Civ. Eng..

[23] Türkmen İ., Bingöl A.F., Tortum A., Demirboğa R., Gül R. (2017). Properties of pumice aggregate concretes at elevated temperatures and comparison with ANN models. Fire Mater..

[24] Nikoo M., Zarfam P., Sayahpour H. (2015). Determination of compressive strength of concrete using Self Organization Feature Map (SOFM). Eng. Comput..

[25] Adeli H. (2001). Neural networks in civil engineering: 1989–2000. Comput.-Aided Civ. Infrastruct. Eng..

[26] Safiuddin M., Raman S.N., Salam M.A., Jumaat M.Z. (2016). Modeling of compressive strength for self-consolidating high-strength concrete incorporating palm oil fuel ash. Materials.

[27] Mansouri I., Kisi O. (2015). Prediction of debonding strength for masonry elements retrofitted with FRP composites using neuro fuzzy and neural network approaches. Compos. Part B Eng..

[28] Mansouri I., Gholampour A., Kisi O., Ozbakkaloglu T. (2016). Evaluation of peak and residual conditions of actively confined concrete using neuro-fuzzy and neural computing techniques. Neural Comput. Appl..

[29] BS (1986). Recommendations for Measurement of Velocity of Ultrasonic Pulses in Concrete.

[30] ASTM C 597-83 (1991). Test for Pulse Velocity through Concrete.

[31] McCann D.M., Forde M.C. (2001). Review of NDT methods in the assessment of concrete and masonry structures. NDT E Int..

[32] Bungey J.H., Millard S.G., Grantham M.G. (2004). Testing of Concretes in Structures.

[33] Bungey J.H., Soutsos M.N. (2001). Reliability of partially-destructive tests to assess the strength of concrete on site. Constr. Build. Mater..

[34] Tharmaratnam K., Tan B.S. (1990). Attenuation of ultrasonic pulse in cement mortar. Cem. Concr. Res..

[35] Ongpeng J., Soberano M., Oreta A., Hirose S. (2017). Artificial neural network model using ultrasonic test results to predict compressive stress in concrete. Comput. Concr..

[36] García-Iruela A., Fernández F.G., Esteban L.G., De Palacios P., Simón C., Arriaga F. (2016). Comparison of modelling using regression techniques and an artificial neural network for obtaining the static modulus of elasticity of Pinus radiata D. Don. timber by ultrasound. Compos. Part B Eng..

[37] Khademi F., Akbari M., Jamal S.M. (2016). Prediction of concrete compressive strength using ultrasonic pulse velocity test and artificial neural network modeling. Romanian J. Mater..

[38] Sheen N.Y., Huang J.L., Le H.D. (2013). Predicting strength development of RMSM using ultrasonic pulse velocity and artificial neural network. Comput. Concr..

[39] Hornik K., Stinchcombe M., White H. (1989). Multilayer feedforward networks are universal approximators. Neural Netw..

[40] Plevris V., Asteris P.G. Modeling of masonry compressive failure using Neural Networks. Proceedings of the OPT-i 2014—1st International Conference on Engineering and Applied Sciences Optimization.

[41] Plevris V., Asteris P.G. (2014). Modeling of masonry failure surface under biaxial compressive stress using Neural Networks. Constr. Build. Mater..

[42] Plevris V., Asteris P. Anisotropic failure criterion for brittle materials using Artificial Neural Networks. Proceedings of the COMPDYN 2015—5th ECCOMAS Thematic Conference on Computational Methods in Structural Dynamics and Earthquake Engineering.

[43] Giovanis D.G., Papadopoulos V. (2015). Spectral representation-based neural network assisted stochastic structural mechanics. Eng. Struct..

[44] Asteris P.G., Plevris V. Neural network approximation of the masonry failure under biaxial compressive stress. Proceedings of the 3rd South-East European Conference on Computational Mechanics (SEECCM III), an ECCOMAS and IACM Special Interest Conference.

[45] Asteris P.G., Plevris V. (2016). Anisotropic Masonry Failure Criterion Using Artificial Neural Networks. Neural Comput. Appl..

[46] Bartlett P.L. (1998). The sample complexity of pattern classification with neural networks: The size of the weights is more important than the size of the network. IEEE Trans. Inf. Theory.

[47] Karlik B., Olgac A.V. (2011). Performance Analysis of Various Activation Functions in Generalized MLP Architectures of Neural Networks. Int. J. Artif. Intell. Expert Syst..

[48] Asteris P.G., Kolovos K.G. (2017). Data on the physical and mechanical properties of soilcrete materials modified with metakaolin. Data Brief.

[49] Delen D., Sharda R., Bessonov M. (2006). Identifying significant predictors of injury severity in traffic accidents using a series of artificial neural networks. Accid. Anal. Prev..

[50] Iruansi O., Guadagnini M., Pilakoutas K., Neocleous K. Predicting the Shear Strength of RC Beams without Stirrups Using Bayesian Neural Network. Proceedings of the 4th International Workshop on Reliable Engineering Computing, Robust Design—Coping with Hazards, Risk and Uncertainty.

[51] Asteris P.G., Kolovos K.G. (2017). Self-Compacting Concrete Strength Prediction Using Surrogate Models. Neural Comput. Appl..

[52] Lourakis M.I.A. (2005). A Brief Description of the Levenberg-Marquardt Algorithm Implemened by levmar. http://www.ics.forth.gr/~lourakis/levmar/levmar.pdf.

